# Nutritional Composition, Antinutritional Factors, and Utilization Trends of Ethiopian Chickpea (*Cicer arietinum* L.)

**DOI:** 10.1155/2021/5570753

**Published:** 2021-05-13

**Authors:** Lamesgen Yegrem

**Affiliations:** Debre Zeit Agricultural Research Centre, EIAR, P.O. Box 2003, Addis Ababa, Ethiopia

## Abstract

Chickpeas are a very important legume crop and have an abundant amount of proteins, carbohydrates, lipids, fibers, and mineral contents. Most of the time, breeders were focused on the yield and the disease resistance criteria parameters for releasing new varieties, but not that much attention is given to the nutritional quality and quantity aspect. So the objective of this review mainly focuses on giving some hints for breeders and nutritionists on nutritional profiles and effects of traditional processing of different Ethiopian chickpea varieties which may be used for variety selection for the new variety trial and new product development, respectively. Chickpeas have many bioactive compounds, important vitamins, and minerals. Besides having nutritional benefits, the consumption of chickpeas always requires some processing as they have many antinutritional factors. Various traditional processes such as soaking, cooking or boiling, germination, roasting, fermentation, and dehulling have their own effects on the availability of nutrients. Chickpeas are used to make many Ethiopian traditional chickpea-based food products such as nifro, kollo, shiro, dabo, mitad shiro, ashuk, boklet, kita, genfo, injera, and shimbra-asa by using different processing methods. Chickpeas have several potential health beneficial effects on some of the important human diseases like cardiovascular diseases, type 2 diabetes, digestive diseases, and cancers. This review summarized that different Ethiopian chickpea varieties have significant differences in the nutritional composition profiles between different varieties grown in Ethiopia and are an excellent source of micronutrients and macronutrients.

## 1. Introduction

Legumes belong to the family *Leguminosae* and consist of pulses, including the dry grains of peas, chickpeas, lentils, peas, beans, and lupines. Production and use of legumes date back to ancient cultures in Asia, the Middle East, South America, and North Africa. They are cultivated throughout the world for their seeds, harvested, and marketed as primary products. Legumes are important food crops due to their high protein and essential amino acid content. Legumes play an important role in the agriculture and diet of many developing countries and are a major source of dietary nutrients for many people and are thus sometimes referred to as the “poor man's meat.” However, their role appears to be limited because of several factors including low protein and starch digestibility, poor mineral bioavailability, and high antinutritional factors [1].

Chickpea (*Cicer arietinum* L.) is the world's third largest legume crop based on the cultivated area [2]. Chickpea is an important pulse crop grown and consumed all over the world, especially in the Afro-Asian countries. There are two major types of chickpea groups: Desi and Kabuli. The Desi types tend to be smaller angular seeds with thick seed coats that range in color from light tan and speckled to solid black. The Desi-type chickpea seed is wrinkled at the peak with brown, light brown, fawn, yellow, orange, black, or green color. The Desi type has a smaller seed than the Kabuli type. The Kabuli types have larger seeds with paper-thin seed coats that range in color from white to pale cream to tan. The Kabuli type is white to cream in color and has a larger seed than the Desi type [3].

In addition to being an important source of protein, chickpea is also reported to be a good source of minerals. This legume supplies larger amounts of calcium and phosphorus than do other legumes and contains more calcium than whole cow's milk (120 mg/100 g) [4]. And the protein quality is considered to be better than other pulses. Chickpea has significant amounts of all the essential amino acids. Starch is the major storage carbohydrate followed by dietary fiber; lipids are present in low amounts, but chickpea is rich in nutritionally important unsaturated fatty acids like linoleic acid and oleic acid. Chickpea has several nutritional and processing problems, such as the presence of antinutrients, prolonged cooking time, and poor digestibility. Its chemical composition is subject to fluctuations, depending on various factors, e.g., cultivar and maturity stage, environment (mostly weather conditions), and agroecology. The major traditional techniques used in the processing of chickpea are cooking/boiling, soaking, dehulling, milling, roasting, germination, and fermentation, which can enhance the bioavailability of micronutrients. The nutritional value of a diet cannot be determined based on the concentration of individual nutrients which are found in the chickpea, but the bioavailability of each nutrient is affected by the interaction between antinutrients and nutrients.

Chickpea is an important ingredient in various dishes and contributes significantly to the basic daily nutritional requirements of a large segment of society in Ethiopia, including used as shiro like lentils, common beans, peas, and faba beans. Pulses have been used for their nutritional qualities for thousands of years [5]. The interest in chickpeas as food and their potential impact on human health have been revived during the past two to three decades. It is also reported that many pulses overcome the risk of chronic diseases and optimize health. Therefore, chickpea is considered a “functional food” along with its role in providing protein and fiber. Chickpea contains different vitamins, minerals, and several bioactive constituents (phenolics, phytates, enzyme inhibitors, and oligosaccharides) that could help to reduce the risk of chronic diseases.

The aim of this study is to contribute to the understanding of the nutritional profiling, different processing methods, and finally traditional food products prepared from chickpeas. As a first step, we provide literature reviews on recent scientific findings on nutritional profiles and traditional processing methods of chickpea. As a second step, we provide evidence on preference valuation of different Ethiopian chickpea-based food products. Finally, we provide an overview and discussion of different health effects and utilization of chickpeas. One objective of these reviews is to relate the findings from different varieties of proximate composition and functional and mineral content to each other and to identify gaps and needs for future research, mainly for breeders to new variety trials and new product developments based on their nutritional characteristics.

### 1.1. Chickpea Production in Ethiopia

Chickpea is an example of a dry bean. Dry beans, by definition, are legumes grown to the mature stage, allowed to dry, and harvested for the seed within the pods [6]. World chickpea production is approximately 9.4 million metric tonnes. Ethiopia is the largest producer of chickpea in Africa, accounting for about 46% of the continent's production during 1994-2006. It is also the fifth largest producer worldwide and contributes about 3.2% to the total world chickpea production [7]. Chickpea, locally known as shimbra, is one of the major pulse crops (including faba bean, field pea, haricot bean, lentil, and grass pea) in Ethiopia, and in terms of production, it is the second most important legume crop after beans. It contributed about 17.6% of the total pulse production during 2014. The total annual average (1999-2014) chickpea production is estimated at about 260 thousand tonnes. The chickpea production and cultivated area are steadily increasing over the years 1999-2014[7].

The average annual growth rate in the area and production showed that the cultivated area under chickpea and the production of chickpea increased by 2.1% and 7.6%, respectively, during the same period. The production growth rate is relatively higher compared to faba beans (5.7%). Grain yield of chickpea has also shown upward trends, particularly starting from the year 2004 and onwards, with an average annual growth rate of 5.9%. Most of the chickpea is cultivated under rain-fed conditions [8].

Chickpea is one of the main annual crops in Ethiopia in terms of both its share of the total cropped pulse area and its role in direct human consumption. In Ethiopia, chickpea is widely grown across the country and serves as a multipurpose crop [9]. Although chickpea is widely grown in Ethiopia, the major producing areas are concentrated in the two regional states Amhara and Oromia. These two regions cover more than 90% of the entire chickpea area and constitute about 92% of the total chickpea production [10]. Chickpea has a capacity to fix soil nitrogen and thus improves soil fertility and saves fertilizer costs in subsequent crops; it improves more intensive and productive use of land, particularly in areas where land is scarce, and the crop can be grown as a second crop using residual moisture; it reduces malnutrition and improves human health especially for the poor who cannot afford livestock products. It is an excellent source of proteins, fibers, complex carbohydrates, vitamins, and minerals, and last but not least, the growing demand in both the domestic and export markets provides a source of cash for smallholder producers.

The leading chickpea-growing countries in the world are India, Pakistan, Mexico, Turkey, Ethiopia, and Myanmar [11], and Ethiopia is the first from other African countries (Table 1). India and Ethiopia have been proposed as secondary centers for the diversity of cultivated chickpea [12]. Plant genetic resources and genetic diversity present in them provide assurance for future genetic progress and insurance against unforeseen threats to agricultural production [13]. The studies of the genetic diversity of plants are very important for developing high-yielding varieties and for maintaining the productivity of such varieties in the plant breeding strategies. In the studies of Ethiopian chickpea morphological characters, the landraces showed considerable variability within and between chickpea populations [14].

### 1.2. Currently Released Chickpea Varieties in Ethiopia

The national chickpea research program was first started in 1972 at Debre Zeit Agricultural Research Centre to increase the production of chickpea. Up to date, there are a total of twenty-nine improved chickpea varieties consisting of 15 Kabuli types and 14 Desi types, which were developed and released in the country Ethiopia by both the national (DZARC) and regional research programs (Table 2).

## 2. Nutritional Composition of Ethiopian Raw Chickpea Varieties

### 2.1. Proximate Composition

Chickpeas (*Cicer arietinum* L.) are staple foods in many countries and play an enhanced role in the diets of vegetarians around the world. Pulses are a primary source of nourishment and, when combined with cereals, provide a nutritionally balanced amino acid composition with a ratio nearing the ideal for humans. Chickpea is a good source of energy, proteins, minerals, vitamins, and fibers and also contains potentially health beneficial phytochemicals.

The moisture content and ash content of different Ethiopian chickpea varieties vary from 5.73 to 12.10% and 2.47 to 3.87%, respectively (Table 3); the ash content was used to indicate the mineral content of chickpea varieties.

The fibers, an indigestible part of the plants in the human small intestine, are classified as soluble and insoluble fibers. The soluble fibers are slowly digested in the colon; in contrast, insoluble fibers, metabolically inert, are subjected to fermentation in the colon inducing intestinal bacterial growth [16]. The total fiber content of Ethiopian chickpea varieties varies from 18 to 20%. The fiber may influence body weight regulation by physiologic mechanisms involving intrinsic, hormonal, and colonic effects. Ultimately, these mechanisms act to decrease food intake by promoting satiation (lower meal energy content) or satiety (longer duration between meals) or by influencing metabolic fuel partitioning (increased fat oxidation and decreased fat storage). Therefore, it is concluded that fiber-rich diets contain nonstarch fruits, vegetables, whole grains, nuts, and legumes and may be effective in the prevention and treatment of obesity in children [17].

The chickpea exhibits higher fat content than other pulses, with a wide genotypic variation. The total lipid concentration of Ethiopian chickpea types ranges from 3.77 to 7.41% (Table 3). The lipid content of foods is often responsible for their flavor, which in the case of chickpea may contribute to its “nutty” taste. Fat of chickpea seeds is characterized by the high content of essential unsaturated fatty acids: linoleic acid (54.7-56.2% mg), oleic acid (21.6-22.2% mg), and linolenic acid (0.5-2.35% mg), as well as saturated fatty acids such as palmitic acid (18.9-20.4% mg) and stearic acid (1.3-1.7% mg) [18].

The protein content of Ethiopian chickpea varieties ranges from 12.02 to 24.91% (Table 3). The amino acid composition of chickpea is well balanced, apart from the limited sulfur amino acids (methionine and cysteine), and is high in lysine. Hence, chickpea is an ideal companion to cereals, which are known to be higher in sulfur amino acids but limited in lysine. The amino acid content is a very important indicator of the nutritional value of foods. Of all the amino acids, nine are essential and must be present in the diet [18]. Unlike animal proteins, plant proteins do not contain these essential amino acids in the required proportions [19]. The essential and nonessential amino acid content is significantly higher in chickpea powder (38.89% and 58.64% of protein, respectively) [20]. Protein-calorie malnutrition is observed in infants and young children in developing countries and includes a range of pathological conditions arising due to lack of proteins and calories in the diet [21]. Malnutrition affects about 170 million people, especially preschool children and nursing mothers of developing countries in Asia and Africa [22]. Pulses provide a major share of proteins and calories in the Afro-Asian diet. Among the different pulses, chickpea is reported to have higher protein bioavailability [23].

Carbohydrate is the major nutritional component in chickpea, with 52.61 to 67.66% in Ethiopian variety type (Table 3). Generally, legumes contain carbohydrate content (60 to 65%), slightly lower than cereals (70-80%). The major classes of carbohydrates are monosaccharides, disaccharides, oligosaccharides, and polysaccharides [19].

Energy is often expressed as gross energy (MJ/kg) or as a caloric value (kcal/100 g) and refers to the amount of energy contained in food. Energy values for Ethiopian chickpea varieties have been reported from 322.58 to 388.10 (kcal/100 g).

The value gaps between different nutritional compositional contents are reported in Debre Zeit Agricultural Research Centre Food Science and Nutrition Annual Report [27], and the nutritional qualities (chemical compositions and mineral contents) of chickpeas were affected by genotypes and environments which are grown or interacted between them (G∗E interaction).

Its chemical composition is subject to fluctuations, depending on various factors, e.g., cultivar and maturity stage, environment (mostly weather conditions), and agroecology. Some reports have also underlined variations in the chemical composition of these chickpeas. These variations can be due to either intrinsic factors (mainly genetics, which is partly responsible for differences between cultivars and varieties) or extrinsic factors, such as storage, types of soil, agronomic practices, climatic factors, and technological treatments [31]. Debre Zeit Agricultural Research Centre Food Science and Nutrition Annual Report (2019) indicated that the samples grown and collected from three different locations have different soil character, annual rainfall, and humidity and altitude areas.

### 2.2. Mineral and Antinutritional Contents

The most important minerals contained in chickpeas are calcium, phosphorus, magnesium, iron, copper, zinc, sodium, and potassium. Most of the seed calcium is located in the seed coat. Therefore, the consumption of whole seed would be useful in calcium-deficient diets. Chickpeas are also a good source of iron. They contain a higher level of iron in comparison with other legumes [32].

An antinutrient is a substance occurring in the diet which acts antagonistically toward one or multiple nutrients, reducing bioavailability. This is usually done through complex formation which reduces nutrient absorption [33]. Tannins are polyphenol components prevalent in food legumes. Studies have shown that tannins interact with proteins, enzymes, or nonenzymes and form tannin-protein complexes, which decrease protein digestibility and protein solubility. Phytate, which is also known as inositol hexakisphosphate, is a phosphorus-containing compound that binds with minerals and inhibits mineral absorption. Phytic acid binds trace elements and macroelements such as zinc, calcium, magnesium, and iron in the gastrointestinal tract, making dietary minerals unavailable for absorption and utilization by the body. The mineral and antinutritional contents of selected Ethiopian chickpea varieties are presented in Table 4.

## 3. Domestic Processing Techniques and Their Effects on the Nutritional Qualities and Antinutritional Contents of Chickpeas

Traditional processing of chickpea is labor-intensive and is mostly done by women, especially in developing countries in Asia and Africa. The major traditional techniques used in the processing of chickpea are cooking/boiling, soaking, dehulling, milling, roasting, germination, and fermentation, which can enhance the bioavailability of micronutrients in plant-based diets by decreasing phytate content and improving overall digestibility and absorption of nutrients (Table 5). Irrespective of the type of food that is prepared from legumes, they are taken through at least more than one process. For example, germination may be followed by boiling, roasting, and further boiling or by steaming and so on. Many of the processes involved in food preparation have beneficial effects. They improve not only taste, aroma, digestibility, and acceptance from consumers but also nutritional quality and reduce unwanted material. However, in developing a product, the food is often subjected to more; we will discuss some of the principles of these technologies and their effects on the nutritional qualities of chickpeas [35].

### 3.1. Soaking

Soaking is often used as pretreatment to facilitate the processing of legumes, and it may last for a short or very long period (20 minutes to 16 hours). Because phytate is water-soluble, a significant phytate reduction can be realized by discarding the soak water. In addition, the action of endogenous phytases contributes to phytate reduction. The temperature and pH value have been shown to have a significant effect on enzymatic phytate hydrolysis during soaking. Part of the inhibitors leaches out during soaking, which will have beneficial effects on health in addition to the reduction in cooking time. Soaking allows the water to disperse in the protein fraction and starch granules which facilitate the protein denaturation and starch gelatinization, which soften the texture of beans.

### 3.2. Cooking/Boiling

Pulses improve the nutritional value due to the decrease or destruction of most antinutritional factors and increased solubility of many nutrients. Soaking pulses before cooking is a common practice. However, changes in the nutritional value will depend on the intensity and duration of heat while cooking, which is influenced by the method used.

### 3.3. Germination/Sprouting

Germination is a process widely used in chickpeas to increase their palatability and nutritional value, particularly through the breakdown of certain antinutrients. The extensive enzymatic activity during the germination process causes the production of essential amino acids and absorbable polypeptides. Germination retains the minerals found in the seeds of chickpea [36]. The germination process is most effective against antinutritional factors in legume seeds; this process lowers the phytate contents in legumes that depend upon the germination method and the type of beans. During germination, the degradation of stored carbohydrates in the seeds by enzymes takes place. This results in significant changes in the physicochemical characteristics of the legumes, including the modification of antioxidant activities [37].

### 3.4. Roasting

Roasting is an essential operation and one of the most frequent processing techniques for seeds [38]. It is intended to increase the palatability of the product, and it significantly promotes the development of color, flavor, texture, and appearance of seeds. Roasting also destroys unwanted microorganisms and inactivates the enzymes that promote deterioration of the product during storage [39]. This treatment allows the preservation of nutrients, as it is a dry treatment compared to the wet cooking that causes leaching. Roasting reduces the levels of oligosaccharides.

### 3.5. Fermentation

Fermentation covers a wide range of microbial and enzymatic processing of foods and ingredients to achieve desirable characteristics such as prolonged shelf life, improved safety, attractive flavor, nutritional enrichment, elimination of antinutrients, and promotion of health. The type of microorganism, the fermentation conditions used, and the starting amount of phytate present in the raw material significantly affect the extent of phytate removal during the fermentation process.

### 3.6. Dehulling

Dehulling involves the removal of the hulls of grain seeds, in this case, legume seeds. The dehulling of legumes results in the reduction of fiber and tannin content and, most importantly, affects the appearance, texture, cooking quality, digestibility, and palatability of the grains [40].

## 4. Chickpea and Chickpea-Based Food Products Commonly Consumed in Ethiopia

Traditionally, chickpea is one of the most favored of all pulses in Ethiopian society. In Ethiopia, the chickpea grain is widely used in different forms as follows: green vegetable, kollo, nifro, dabo, genfo (porridge), kita, shimbra-asa, boklet, kik, mitad shiro, and shiro which is used to prepare the so-called “wot” (sauces) eaten with Ethiopian injera. Discussed below are the food products (Table 6).

## 5. Health-Improving Effects of Chickpea

Chickpea consumption is reported to have some physiologic benefits that may reduce the risk of chronic diseases and optimize health. Therefore, chickpeas could potentially be considered a “functional food” in addition to their accepted role of providing proteins and fibers. Chickpea is a relatively inexpensive source of different vitamins, minerals, and several bioactive compounds [41]. These compounds included certain antinutritional compounds, phenolic compounds including flavonoids, phenolic acids, and isoflavones, bioactive peptides with antioxidant, anticancerous, and antihypertensive properties, nondigestible carbohydrates such as dietary fibers and resistant starch, carotenoids, and phytosterols. These could aid in potentially lowering the risk of chronic diseases.

Chickpea seed oil contains different sterols, tocopherols, and tocotrienols [42, 43]. These phytosterols are reported to exhibit antiulcerative, antibacterial, antifungal, antitumoric, and anti-inflammatory properties coupled with a lowering effect on cholesterol levels [44]. Chickpea is reported to have higher levels of carotenoids (explained above) than “golden rice,” and it could be potentially used as a source of dietary carotenoids. Carotenoids like lutein and zeaxanthin, the major carotenoids in chickpea seeds, are speculated to play a role in senile or age-related macular degeneration. Carotenoids are reported to increase natural killer cell activity [45]. Vitamin A, a derivative of *β*-carotene, is important in several developmental processes in humans like bone growth, cell division/differentiation, and most importantly vision.

## 6. Conclusion

Nutritional composition, antinutritional factors, and utilization trends of Ethiopian chickpea (*Cicer arietinum* L.) were reviewed. The chemical constituents of chickpea seeds including both the nutritional and antinutritional factors have been studied by several workers. Although there appears to be a large variation among cultivars, few efforts have been made to show the effect of the environment on such constituents. An attempt should be made to establish whether the phenomenal differences are consistent across a variety of environments. This information would also be useful in implicating the dietary potential of chickpea in human nutrition. From different scholar outputs, some antinutritional factors are presented in chickpeas but they can be reduced by using different traditional household processing methods. This review provides an insight into different traditional processing methods which are used to produce local chickpea-based food products.

According to this review, the researcher recommends that based only on the protein content of Ethiopian chickpea varieties, the Arerti variety scored the highest and is recommended for consumers and can be used for different protein-enriched complementary food products. The possibility of utilizing chickpea for the preparation of Ethiopian traditional food products like shiro-wot and kik-wot like by heat treatments and their effects on nutritional quality need to be explored. Further research is needed on the characterization of the nutritional compositions of different chickpea varieties on the untouched parts like the amino acid profiling and locally produced Ethiopian chickpea-based food products by considering promising Ethiopian chickpea varieties of the country and optimizations of different processing parameters for locally produced chickpea-based food products. Factors such as growing conditions, chemical composition, and storage should be studied in relation to chickpea nutritional quality of chickpeas grown in Ethiopia.

## Figures and Tables

**Table 1:** Top chickpea-producing countries in the world from 2013 to 2017.

Country	Production (tonnes)	Region
India	41,827,500	South Asia
Australia	4,876,693	Australia
Myanmar	2,790,562	South Asia
Turkey	2,341,000	West Asia
Ethiopia	2,307,096	Africa
Pakistan	2,145,445	South Asia
Iran	1,199,901	West Asia
Russia	998,293	Europe
USA	963,523	North America

Source: Food and Agriculture Organization (FAO) [11].

**Table 2:** Chickpea varieties released in Ethiopia (1974-2019).

Variety	Type	Origin	Year of release
DZ-10-4	Kabuli	Ethiopia	1974
DZ-10-11	Desi	Ethiopia	1974
Dubie	Desi	Ethiopia	1978
Mariye	Desi	ICRISAT	1985
Worku	Desi	ICRISAT	1994
Akaki	Desi	ICRISAT	1995
Arerti	Kabuli	ICARDA	1999
Shasho	Kabuli	ICARDA	1999
Habru	Kabuli	ICARDA	2004
Chefe	Kabuli	ICARDA	2004
Ejere	Kabuli	ICARDA	2005
Teji	Kabuli	ICARDA	2005
Kutaye	Desi	ICRISAT	2005
Mastewal	Desi	ICRISAT	2006
Fetenech	Desi	ICRISAT	2006
Yelbie	Kabuli	ICRISAT	2006
Natoli	Desi	ICRISAT	2007
Acos Dubie	Kabuli	Mexico	2009
Minjar	Desi	ICRISAT	2010
Kasech	Kabuli	ICRISAT	2011
Akuri	Kabuli	ICRISAT	2011
Kobo	Kabuli	ICRISAT	2012
Dalota	Desi	ICRISAT	2013
Teketay	Desi	ICRISAT	2013
Dimtu	Desi	ICRISAT	2016
Hora	Kabuli	ICARDA	2016
Dhera	Kabuli	ICARDA	2016
Koka	Kabuli	ICRISAT	2019
Geletu	Desi	ICRISAT	2019

Source: Asnake and Dagnachew [15].

**Table 3:** Chemical composition of some of Ethiopian raw whole chickpea varieties.

Chickpea varieties	Ash (%)	Moisture (%)	Crude protein (%)	Crude fat (%)	Crude fiber (%)	Carbohydrate (%)	Gross energy (kcal/100 g)	Reference
Natoli	3.77		18.71	6.97	5.81	55.90	361.13	[24]
Arerti	3.87	9.07	21.78	7.41	4.71	53.16	366.46	

Habru	2.49	6.96	20.03	6.88	4.23	59.41		[25]
Local-Desi	3.43	7.00	21.90	4.61	6.97	56.10		

DZ-10-11	2.63	7.60	16.73	5.88	5.88	61.22	364.69	[26]

Habru	3.16	7.52	20.92	7.01	5.09	56.30	371.91	[24]
Mastewal	2.97	7.27	19.88	6.02	8.19	55.67	356.38	
Local	3.43	5.73	19.57	3.77	16.91	52.61	322.58	

Natoli	2.98-3.47	10.28-11.32	15.93-20.21	4.61-5.75		61.94-66.84	366.98-385.33	[27]
Ejeri	2.69-3.29	10.41-11.80	16.19-20.61	5.24-7.01		61.45-64.87	371.91-388.10	
Teketaye	2.71-3.25	10.97-11.38	14.01-19.62	4.89-6.71		65.00-67.50	368.20-383.54	
Hora	2.47-3.36	10.08-11.84	16.07-20.38	4.95-6.93		62.45-65.57	370.75-380.92	
Dera	3.00-3.35	10.03-12.10	18.43-21.82	4.94-6.55		59.07-63.39	355.80-376.30	
Arerti	2.73-3.26	10.46-11.23	15.02-21.73	5.83-7.34		59.17-65.05	371.51-381.92	
Dimtu	2.77-3.29	10.40-11.83	14.12-21.12	4.54-6.27		60.71-67.66	364.30-378.66	
Habru	2.60-3.15	10.21-11.07	16.49-21.26	6.07-7.09		60.48-65.40	373.81-380.74	
19 (candidate)	2.88-3.45	10.87-11.26	15.41-21.58	5.38-6.09		60.13-66.25	368.99-375.41	
Shasho	2.73-3.64	10.45-11.55	16.01-19.89	5.27-7.32		60.52-66.57	365.60-383.86	
24 (candidate)	2.72-3.12	10.58-11.18	14.67-18.70	5.77-7.03		63.68-65.90	372.70-381.92	

Desi type	2.71	11.19	21.76	4.48	2.85	57.01	355.40	[28]
Arerti	3.68	10.65	24.91	5.21	1.42	54.13	363.05	

Kabuli variety	2.70	7.69	21.07	5.94	6.56	62.60	388.12	[29]

Chickpea flour	3.40	8.00	19.40	5.80	4.80	63.40	383.00	[30]

**Table 4:** Mineral and antinutritional contents of some of Ethiopian raw whole chickpea varieties.

Varieties	Iron (mg/100 g)	Calcium (mg/100 g)	Phosphorus (mg/100 g)	Zinc (mg/100 g)	Phytate (mg/100 g)	Tannin (mg/100 g)	Reference
Habru	6.47	147.47	375.24	3.69	60.20	23.23-29.56	[24]
Mastewal	4.04	146.48	228.24	2.05	58.59-59.99	103.41	
Local	4.99	400.78	216.35	3.04	63.28	62.12-68.32	

Natoli	1.07	126.73	—	0.71	86.54-88.28	0.16 (%)	[34]

DZ-10-11	6.79	207.40	298.16	3.95	97.46	175.23	[26]

Natoli	5.05-9.84	153.06-277.98	301.01-545.37	1.57-3.00	—	—	[27]
Ejeri	5.13-9.45	143.31-272.47	345.33-560.60	1.92-3.04	—	—	
Teketaye	5.00-9.98	163.49-253.78	284.74-42.57	1.59-3.09	—	—	
Hora	5.03-8.96	175.84-277.67	329.16-472.67	2.05-3.33	—	—	
Dera	5.41-9.06	150.65-208.74	332.76-535.48	2.18-2.87	—	—	
Arerti	4.93-9.21	134.99-284.35	345.56-560.76	2.18-2.94	—	—	
Dimtu	5.65-9.36	138.06-212.57	276.95-549.51	1.51-2.84	—	—	
Habru	5.19-8.84	128.80-234.32	300.83-516.34	1.87-2.85	—	—	
19 (candidate)	5.27-9.44	125.73-242.10	341.81-478.39	1.93-2.51	—	—	
Shasho	5.04-9.38	121.86-217.93	307.19-542.23	1.89-2.88	—	—	
24 (candidate)	4.70-9.80	132.53-214.69	309.09-524.62	1.84-2.48	—	—	

Arerti	—	—	—	4.15	—	—	—
Habru	—	—	—	3.76		—	
Mastewal	—	—	—	3.41	—	—	
Natoli	—	—	—	4.02	—	—	

Kabuli variety	6.29	143.25	—	2.55	94.76	162.82	[29]

Chickpea flour	6.80	117.00	330.00	—	—	—	[30]

**Table 5:** Summary of effects of some traditional processes in nutritional composition and mineral and antinutritional contents of different Ethiopian chickpea varieties.

Processing methods	Moisture (%)	T.ash (%)	C.protein (%)	C.fat (%)	C.fiber (%)	CHO (%)	Energy (kcal/100 g)	Ca	P	Fe	Zn	Phytate	C.tannin	Reference
Whole, fresh, raw local chickpea	77.60	0.80	4.60	1.00	4.20	16.00	91.40	52.00	73.00	6.30	—	—	—	[30]
Whole, fresh, boiled local chickpea	59.30	1.40	7.30	2.00	5.50	30.00	167.00	83.00	101.00	3.40	—	—	—	
Whole, fresh, roasted local chickpea	42.70	1.40	10.10	2.90	8.80	42.90	238.10	150.00	162.00	7.40	—	—	—	
Whole, dried local chickpea	10.50	2.30	10.40	4.70	9.90	72.10	372.30	200.00	238.00	7.00	—	—	—	
Dried, roasted, boiled local chickpea	29.40	3.30	13.00	4.00	9.40	50.30	289.20	172.00	174.00	5.70	—	—	—	
Germinated, boiled local chickpea	27.60	2.10	13.80	4.10	9.90	52.40	301.70	170.00	210.00	5.20	—	—	—	
Germinated, raw local chickpea	49.70	1.30	9.40	2.80	0.70	36.80	210.00	147.00	135.00	3.70	—	—	—	
Chickpea sauce without chili (*shimbra wot*, *allicha*)	79.20	1.10	2.30	8.90	0.70	8.50	123.30	23.00	40.00	2.30	—	—	—	
Chickpea split sauce without chili (*shimbra kik*-*wot*)	68.60	1.20	4.80	4.30	1.60	21.10	142.30	35.00	68.00	2.10	—	—	—	

Raw DZ-10-11	7.60	2.62	16.73	5.87	5.87	61.22	364.69	207.40	298.16	6.79	3.95	97.46	175.23	[26]
Boiled DZ-10-11	8.86	2.41	13.87	4.77	4.57	65.32	359.72	191.43	265.36	4.19	3.21	97.17	174.37	
Wet roasted DZ-10-11	9.37	2.30	13.82	5.20	5.19	64.12	359.26	178.52	260.62	4.04	2.66	91.93	160.10	
Dry roasted DZ-10-11	5.91	2.59	14.08	6.07	3.36	68.67	382.95	162.60	252.19	4.00	2.83	96.00	174.90	
Germinated DZ-10-11	6.90	2.31	15.39	4.26	5.60	65.55	362.10	170.75	294.26	5.44	3.15	72.07	99.26	
Fermented at 24 h DZ-10-11	6.82	2.53	15.71	5.72	4.87	64.34	371.80	137.12	271.06	5.92	3.59	84.26	169.90	

Raw Kabuli varieties	7.69	2.70	21.07	5.94	6.56	62.60	388.12	143.25	—	6.29	2.55	94.76	162.82	[29]
Soaked Kabuli	8.06	2.47	20.23	6.08	4.53	63.16	388.30	140.00	—	6.04	2.53	81.29	81.75	
Germinated Kabuli	7.30	2.45	20.92	5.85	4.23	63.48	390.26	137.00	—	5.88	2.22	68.08	35.61	

Raw Natoli variety	—	3.77	18.71	6.97	5.81	55.90	361.13	—	—	—	—	—	—	[34]
Dry roasted Natoli	—	3.44	12.51	6.94	3.93	68.52	386.54	—	—	—	—	—	—	
Dehulled Natoli	—	3.21	22.62	8.48	2.43	56.52	392.86	—	—	—	—	—	—	
Soaked Natoli	—	3.67	15.15	7.08	5.16	62.24	373.29	—	—	—	—	—	—	
Germinated Natoli	—	3.60	20.21	7.39	5.32	54.85	366.75	—	—	—	—	—	—	
Boiled Natoli	—	3.48	19.91	7.43	4.91	53.34	360.20	—	—	—	—	—	—	

Note: C.tannin = condensed tannin; C.fiber = crude fiber; T.ash = total ash; C.protein = crude protein; C.fat = crude fat.

**Table 6:** Different Ethiopian traditional chickpea-based food products and their preparation mechanisms for utilization.

No.	Product name	Characteristics, processing conditions, and mode of utilization of chickpea-based food products
1	Green immature seeds	The pods are opened by hand, and seeds are eaten green. Green immature chickpea pods harvested a week or two before they mature are consumed as snacks. The green seeds separated from pods have less starch and protein and more sugar than the mature form.
2	Boklet	It is a sprouted whole seed. In this process, chickpea seeds are washed and soaked in water for 5–6 h at room temperature. After washing, all the seeds are kept in a fine cotton cloth for 24–48 h at room temperature for sprouting. During this time, healthy seeds will start to germinate. Germinated and sprouted seeds are washed along with salt and consumed as breakfast.
3	Kollo	It is prepared as follows. The chickpea seeds were soaked or cooked for two days. The soaked seeds were roasted using heat until they will become ready to eat. This popular local snack, *kollo*, is consumed either alone or in mixed cereals with different legume families, but most of the time, wheat or barley *kollo* from cereals was mixed with that of chickpea.
4	Nifro	The chickpea grains were soaked for two days at room temperature with water; the soaked chickpea grains were cooked by adding enough water using heat until they will become ready to eat. And they can be mixed with cereals, commonly wheat, and then the snack is eaten after the addition of salt.
5	Shiro	The raw chickpea seed was soaked overnight. After soaking, chickpea grains were dried in sunlight and roasted, then crashed into single cotyledons and milled to prepare the so-called *shiro* (Ethiopian roasted chickpea which is used to prepare *wot* eaten with injera). Flour from roasted, dehulled, and spiced chickpea is used as a thickener, and the mixture is allowed to simmer. This is called shiro-wot. Wot is always served with injera, the leavened bread made from cereals.
6	Kik-wot	The raw chickpea seed was soaked overnight. After soaking, chickpea grains were dried in sunlight and roasted, then used as whole, shelled, and split to produce *dhal*. The *wot* from the split seed of chickpea was called kik-wot.
7	Genfo (porridge)	Traditionally, in many parts of Ethiopia, there is a habit of preparing genfo for an expectant mother. For this purpose, different cereals, mainly wheat and barley grain, were mixed with legumes like chickpea flour. In addition, genfo is also considered appropriate complementary food for children aged between 6 months and 24 months.
8	Shimbra-asa (chickpea fish)	A popular and unique dish for fasting days is prepared from chickpea as follows. Using dehulled chickpea flour, unleavened small pieces of bread of different shapes are baked on a clay griddle. The same basic sauce mentioned above is prepared, and the bread is dropped into the boiling sauce and allowed to simmer. It is called *shimbra*-*asa wot*.
9	Mitad shiro	It is a thick, relatively drier paste made on a clay pan. Chickpea flour was mixed with water with small salt and spry on heated *mitad* and cooked.
10	Kita	Kita is dry, thin, flatbread with a chew consistency similar to a chewy pretzel. To make kita, the flour is mixed (wheat and chickpea) with water and kneaded by hand with a pinch of salt to make thick unfermented dough. It is then baked immediately on both sides using a clay pan (*mitad*) or iron pan (*biret*-*mitad*). When one side is baked enough, it is turned inside out so as to allow the other side to bake. Kita is relatively thicker and harder bread but smaller in size (about the size and thickness of a pizza base) compared with *injera*.
11	Infant food	Chickpea is blended with cereals and/or other legumes for preparing foods for infants and young children using traditional food products like chickpea-incorporated maize-based flatbread for preschool children, chickpea stew, and chickpea and corn salad.
12	Injera	*Injera* is thin and fermented Ethiopian traditional bread made from flour, water, and starter (*ersho*), which is a small portion from previously fermented dough. It is the most widely consumed food because it accompanies almost all traditional dishes in Ethiopia and is served with sauces. So chickpea is used to prepare *injera* by being mixed with other cereals.
